# Multi-Sensor Data Fusion Using a Relevance Vector Machine Based on an Ant Colony for Gearbox Fault Detection

**DOI:** 10.3390/s150921857

**Published:** 2015-08-31

**Authors:** Zhiwen Liu, Wei Guo, Zhangchun Tang, Yongqiang Chen

**Affiliations:** School of Mechatronics Engineering, University of Electronic Science and Technology of China, Chengdu 611731, China; E-Mails: guo.w@uestc.edu.cn (W.G.); tangzhangchun@uestc.edu.cn (Z.T.); chenyongqiang@uestc.edu.cn (Y.C.)

**Keywords:** gearboxes, multi-senor data fusion, relevance vector machine, ant colony optimization, fault detection

## Abstract

Sensors play an important role in the modern manufacturing and industrial processes. Their reliability is vital to ensure reliable and accurate information for condition based maintenance. For the gearbox, the critical machine component in the rotating machinery, the vibration signals collected by sensors are usually noisy. At the same time, the fault detection results based on the vibration signals from a single sensor may be unreliable and unstable. To solve this problem, this paper proposes an intelligent multi-sensor data fusion method using the relevance vector machine (RVM) based on an ant colony optimization algorithm (ACO-RVM) for gearboxes’ fault detection. RVM is a sparse probability model based on support vector machine (SVM). RVM not only has higher detection accuracy, but also better real-time accuracy compared with SVM. The ACO algorithm is used to determine kernel parameters of RVM. Moreover, the ensemble empirical mode decomposition (EEMD) is applied to preprocess the raw vibration signals to eliminate the influence caused by noise and other unrelated signals. The distance evaluation technique (DET) is employed to select dominant features as input of the ACO-RVM, so that the redundancy and inference in a large amount of features can be removed. Two gearboxes are used to demonstrate the performance of the proposed method. The experimental results show that the ACO-RVM has higher fault detection accuracy than the RVM with normal the cross-validation (CV).

## 1. Introduction

Gearboxes are key components in mechanical transmission systems and widely used in industry. It is reported that the breakdowns of the transmission machinery resulted from the gearbox failures account for 80% and the malfunctions of the gearbox are mostly caused by the gear and bearing faults [1]. Once there exists a fault in the gearbox, it gradually accumulates and finally causes unwanted fatal breakdowns, production losses and human casualties. Therefore, it is necessary and critical that the real-time condition monitoring and fault diagnosis for gearboxes should be performed to early identify their failure information and then reduce the corresponding losses. For gearbox fault diagnosis, intensive study has been done in the past decades and vibration-based analysis is the most commonly used method and also proved to be efficient in various real applications. Many classical methods, such as Fourier transform (FT) [2,3], wavelet transform (WT) [4,5], and signal modulation and demodulation [6,7], *etc*. have been applied to vibration signals to extract the status of gearbox for fault diagnosis.

Nowadays, the trend for the ever-increasing complexity of these systems is to apply the condition based maintenance (CBM) [8]. Considering that the CBM heavily relies on information provided by sensors, invalid or inaccurate measurement data from only one sensor, for example, working temperature and noise disturbance may result in inaccurate conclusion and inappropriate actions. Therefore, the performance and reliability of sensors must been taken into consideration, and multi-sensor data fusion can potentially improve the detection capabilities and probability that any damage is detected. In standard sensor fusion architecture, data from more than one sensors and related information from associated databases are combined to improve the accuracy levels as well as to provide more specific inferences than what could be obtained by the use of a single sensor [9].

Condition-based maintenance for complex process can be divided into two categories: model-based and data-driven techniques, the former of which has been proven to be inadequate in addressing the stochastic nature of the plant. Recently, some popular data-driven methods, such as artificial neural networks (ANN) [10,11,12], fuzzy logic [13,14] and Support vector machine (SVM) [15]. ANN and fuzzy logic are based on an empirical risk minimization principle and have the limitations, such as local optimal solution, low convergence rate, obvious “overfitting”, and especially poor generalization when the number of fault samples is limited [16]. SVM proposed by Vapnik [17] is advance in handling the problems with small sample size and nonlinear approximations based on statistical learning theory. It adopts the structure risk minimization principle, which avoids local minimum and effective solves the overfitting and assures good generalization ability and better classify accuracy. The special predominance of SVM in resolve limited samples, non-linear function and multidimensional pattern recognition make it become a kind of excellent machine learning method. Despite the fact that the SVM classifier provides successful results, it still has open problems to be solved [18], such as the fact that predictions are not probabilistic and the kernel function must satisfy Mercer’s condition, that is, it must be a positive definite continuous symmetric function. It requires estimation of the penalty factor *C* [19]. The number of the found support vector is sensitive to the given error bound and grows linearly with the size of the training set.

Compared with SVM, the relevance vector machine (RVM) proposed by Tipping [18] is also a kernel-based learning algorithm but not restricted by Mercerʼs condition. It is based on a linear model Bayesian formulation with an appropriate prior that results in sparse representation, and offers good generalization performance using the sparse representation that contains few non-zero basis functions, called relevant vectors (RVs). Therefore, it is viewed as a probabilistic version or Bayesian extension of SVM [20,21]. Till now, RVM has attracted much attention for nonlinear classification [22] and prediction [23]. 

To obtain high accuracy of fault detection by the use of RVM, one of its problems, *i.e*., the choice of parameter of kernel function, must be solved because it greatly influences its integrative performance [24]. A possible solution for this is to employ normal optimization algorithms, such as the cross-validation. However, one of limitations for the cross-validation (CV) method is that it consumes important resources, especially CPU time. The Ant Colony Optimization (ACO) algorithm was first proposed as a novel heuristic approach for difficult combinatorial optimization problems [25]. The principle of ACO algorithm is to find the shortest route between the food source and their nest. The most important is that the parallelism and distributional characteristic ensure the capacity of processing massive data, which is not high to CPU and memory request.

To summarize, in this paper, we introduce a novel intelligent fault detection of multi-sensor data fusion using relevance vector machines (RVMs) with ant colony optimization (ACO) algorithm is applied with experimental validation to the health monitoring of gearbox.

The rest of the paper is organized as follows. In Section 2, RVM optimized by ACO will be presented. In Section 3, the features extraction method will be briefly introduced. In Section 4, the multi-sensors data fusion strategy will be described. Section 5 will discuss the experimental results. Finally, some conclusions are presented in Section 6.

## 2. Theoretical Background

### 2.1. RVM Classifier

RVM based on a Bayesian framework is originally derived for binary classification [18] and has the same functional form as the SVM, which provides probabilistic interpretation of its output. It calculates relevance vectors and weights through maximizing a marginal likelihood.

In supervised learning, there is a set of examples ${\left\{\left(x_{i},t_{i}\right)|x_{i}\in R^{N},\;t_{i}\in \left\{-1,1\right\}\right\}}_{i=1}^{N}$, where $x_{i}$ is an input vector, and $t_{i}$ is a label of $x_{i}$. The functional relationship between $x_{i}$ and $t_{i}$ is given as follows:

(1) $$t_{i}=\sum_{i=1}^{N}w_{i}K\left(x,x_{i}\right)+w_{0}$$

where $w_{i}$ is weight vector; $K\left(x,x_{i}\right)$ is a kernel function and $w_{0}$ is bias. 

Considering scalar-valued target functions only, it follows the standard probabilistic formulation. Assume that the targets for the RVM learning model are class labels from the examples with additive noise.

(2) $$t_{i}^{\prime }=t_{i}+{\text{ε}}_{i}=\sum_{i=1}^{N}w_{i}K\left(x,x_{i}\right)+w_{0}+{\text{ε}}_{i}$$

where ${\text{ε}}_{i}$ is the *i*th in dependent sample from a noise process, which is further assumed to be mean-zero Gaussian with variance ${\text{γ}}^{2}$ . In this paper, since that Gaussian radial basis function (RBF) $K\left(x,x_{i}\right)=\exp\left(-{\left\|x-x_{i}\right\|}^{2}/\left(2{\text{γ}}^{2}\right)\right)$ has a powerful nonlinear processing capability, it is selected as the kernel function, in which γ is the width factor to control the sensitivity of the kernel.

For binary classification problems, let the vector X denote the input to be classified, and the scalar t denotes its class label. Using the generalization linear model and the logistic sigmoid function $\text{σ}\left(y\right)=1/\left(1+e^{-y}\right)$, the likelihood can be written as:

(3) $$P\left(t|W\right)=\prod_{i=1}^{n}\text{σ}{\left\{y\left(X_{i};W\right)\right\}}^{t_{i}}{\left[1-\text{σ}\left\{y\left(X_{i};W\right)\right\}\right]}^{1-t_{i}}$$

where the targets $t_{i}\in \left\{0,1\right\}$. The weights W cannot be analytically obtained, and so are the closed-form expression for either the weight posterior p(W|t,α) or the marginal likelihood P(t|W), with a hyper-parameter vector α. The weights cannot be analytically obtained, and therefore, a Laplace’s approximation procedure is used [26]:

(1) Due to p(W|t,α)∝P(t|W)p(W|α), this is equivalent to finding the maximum of α:

(4) $$\begin{array}{l} \log\left\{P\left(t|W\right)p\left(W|\text{α}\right)\right\}=\sum_{i=1}^{n}\left[t_{i}\;\log y_{n}+\left(1-t_{n}\right)\log\left(1-y_{n}\right)\right]\\ \;\;\;\;\;\;\;\;\;\;\;\;\;\;\;\;\;\;\;\;\;\;\;\;\;\;\;\;\;\;\;\;\;\;\;-\frac{1}{2}W^{T}AW \end{array}$$

(2) Laplace’s method is simply a quadratic approximation to the log-posterior around its mode. Equation (4) is differentiated twice, and then gives:

(5) $$\nabla_{W}\nabla_{W}\log P\left(W|t\right){|}_{W_{MP}}=-\left(\Phi^{T}B\Phi +A\right)$$

where $B=\text{d}iag\left({\text{β}}_{0},{\text{β}}_{1},...,{\text{β}}_{n}\right)$ is a diagonal matrix with ${\text{β}}_{i}=y_{i}\left(1-y_{i}\right)$, and Φ denotes the Gaussian cumulative distribution function.

(3) The hyper-parameter is updated using the following equation:

(6) $${\text{α}}^{new}=\frac{1-{\text{α}}_{i}\sum_{ii}}{W_{MP}^{2}}$$

where $\sum_{ii}$ the *i*th diagonal element of the covariance $\sum ={\left(\Phi^{T}B\Phi +A\right)}^{-1}$ and is similar to $W_{MP}=\sum \Phi^{T}Bt$.

To solve the multi-class problem, the original formulation of RVM may be extended to multi-class classification by generalizing Equation (3) to the multi-nominal form:

(7) $$P\left(t|W\right)=\prod_{i=1}^{n}\prod_{k}^{m}\text{σ}{\left\{y_{k}\left(X_{i};W_{k}\right)\right\}}^{t_{i\;k}}$$

where *m* is the number of classes, $t_{ik}$ is the indicator variable for case *i* to be a member of class *k* and $y_{k}$ is the predictor for class *k*. Here, a true multi-class likelihood can be stated as:

(8) $$P\left(t|W\right)=\prod_{i=1}^{n}\prod_{k}^{m}\text{σ}{\left\{y_{k};y_{1},y_{2},...,y_{k}\right\}}^{t_{i\;k}}$$

### 2.2. RVM Parameter Optimization by ACO

The Ant Colony Optimization (ACO), proposed by Dorigo *et al*. [25] and inspired by the behavior of ants finding paths from their nest to a food source. In ACO, by means of both iterative and parallel process, each ant builds a solution using two types of local information: specific problem information and information added by the ants during previous iterations of the algorithm, which are summarized in a function called pheromone. For more details and discussions of the principles of the ACO algorithm, readers can refer to [25,27].

**Figure 1:**
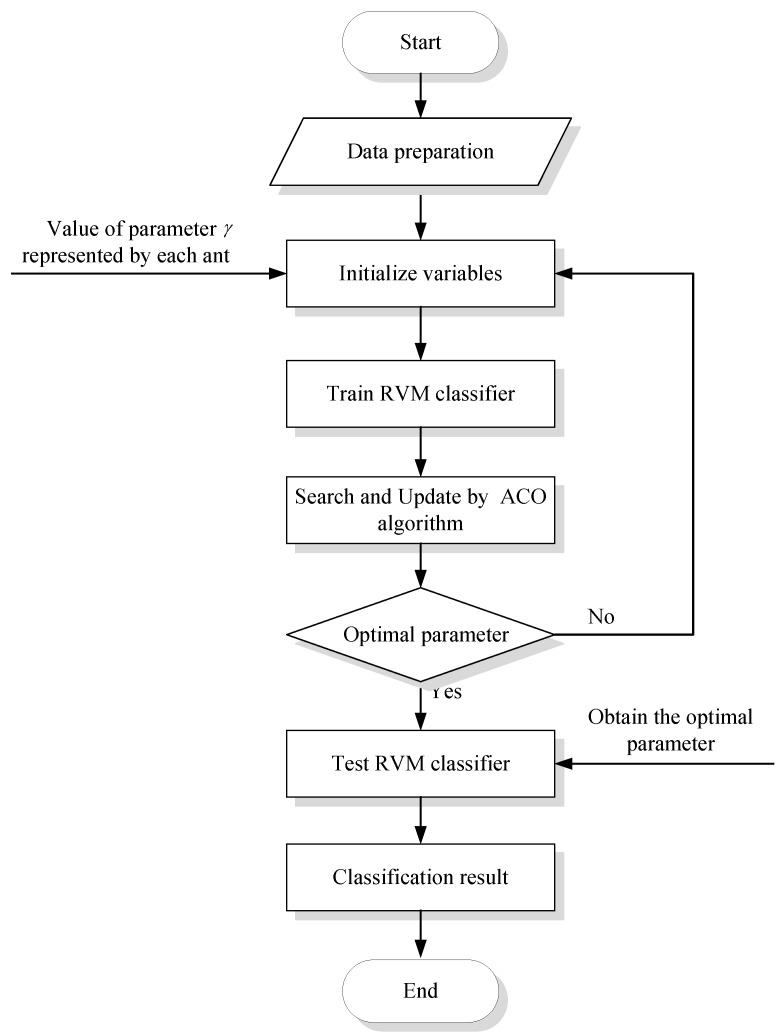
ACO-based parameter optimization of RVM overview.

In the RVM, the width factor γ of the RBF kernel is a user-determined parameter which plays an important role in the classification capability. To precisely establish an ACO-based optimization method for parameter optimization of RVM, the following main steps as shown in Figure 1 must be proceeded. What follows is the detailed explanation of steps for parameter optimization of RVM with ACO algorithm.

***Step 1***: Data preparation. Training, testing samples are represented as Tr and Te, respectively.

***Step* 2**: Initialize variables.

(1) Firstly, assign a random value of the width coefficient γ for RVM to each ant;

(2) According to the assigned initialization parameter (γ), the training sets can be learned using RVM to find the parameters that minimize the diagnosis error, the minimize error is based on the classification accuracy of a RVM classifier, which is as follows:

(9) $$minimize\;T=1-\frac{y_{t}}{y_{t}+y_{f}}$$

where $y_{t}$ and $y_{f}$ represent the numbers of true and false classification samples, respectively.

***Step* 3**: Search and Update.

(1) Initialize parameters and variables of the ACO algorithm. In addition, calculate the grid interval of each parameter after meshing the parameters into N grids.

(10) $$h_{j}=\left(v_{j}^{upper}-v_{j}^{lower}\right)/N,\left(j=1,\cdots ,m\right)$$

where $v_{j}^{upper}$ and $v_{j}^{lower}$ denote the upper and lower limit of a parameter of RVM respectively. $h_{j}$ denotes the parameter interval after meshing, and m is the number of parameters for optimizing.

(2) Rule of state transition and state updating 

In the process of the search, the ants build solutions by applying a probabilistic decision policy to move through adjacent states. The state transition rule is presented as follows:

(11) $$P_{ij}=\frac{\tau_{ij}}{\sum_{i=1}^{N}\tau_{ij}}$$

where $p_{ij}$ is state transition probability, ${\text{τ}}_{ij}$ is artificial pheromone quantity. The $p_{ij}$ expresses the state transition probability of an ant shifting from city i to city j.

The ants select parameter combinations according to the above state transition rule. After training the RVM classifier with the parameters the ants selected, the algorithm evaluates each parameter combination by calculating minimize error according to Equation (9).

The state updating rule is performed only after all the ants have developed their respective solutions. The pheromone level is incremented by applying the state updating rule:

(12) $${\text{τ}}_{ij}^{new}=\left(1-\text{ρ}\right)t_{ij}^{old}+\frac{Q}{e^{T}}$$

where T is the value of minimize error in Equation (9), ρ is evaporation coefficient, and Q is pheromone intensity. The state updating rule intends to provide greater amount of pheromone to the solution set that produces fewer classification errors, which would make them more attractive for the future ants to select.

(3) Find out the corresponding subscript of the node with maximal pheromone quality and diminish the parameter scope:

(13) $$v_{j}^{lower}\leftarrow v_{j}^{lower}+\left(m_{j}-\Delta \right)*h_{j}$$

(14) $$v_{j}^{upper}\leftarrow v_{j}^{upper}+\left(m_{j}+\Delta \right)*h_{j}$$

where Δ is a coefficient. The ants search in the neighborhood of the node with maximal pheromone until to the current iteration of the algorithm.

(4) Stop condition checking. The three steps are repeated until the grid interval $h_{j}$ is less than predefined precision, which is the terminal condition of the optimization algorithm. Finally, the optimal parameters are obtained as follows:

(15) $$v_{j}^{*}=\left(v_{j}^{lower}+v_{j}^{upper}\right)/2\begin{array}{c} , \end{array}\;j=1,\cdots ,m$$

where $v_{j}^{*}$ denotes the obtained optimal parameter values of train RVM classifier. Otherwise, return to ***Step* 2** to conduct the next iteration.

***Step* 4**: According to have obtained the parameter γ of train RVM classifier in ***Step 3***, testing samples are inputted to the test RVM classifier to calculate the classification results.

## 3. Features Extraction

Feature extraction plays a key step in multi-sensor data fusion for gearbox fault detect, which is the determination of a feature vector from a pattern with minimal loss of important information. Therefore, it is extremely important that the fault feature vector correctly and efficiently represents the fault characteristics hidden in the raw signal, which also directly affects the classification results and diagnostic accuracy. 

### 3.1. Signal Preprocessing Based on EEMD

When a gearbox with fault(s) is operating, the generated vibration signals are very complicated with the features of non-linear and non-stationary processes. To effectively extract fault features from the raw vibration signals, the signals are preprocessed using ensemble empirical mode decomposition (EEMD), which is suitable for nonlinear signal analysis. EEMD is a noise-assisted data analysis method, and it is a substantial improvement of EMD, which can solve the problem of mode mixing in EMD [28].

**Figure 2:**
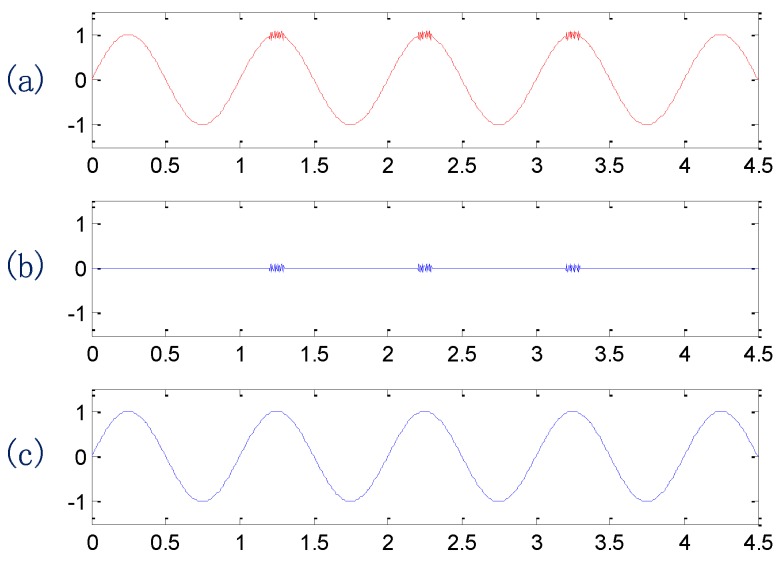
The simulation signal and its two components: (**a**) the simulation signal; (**b**,**c**) the two components.

A simulated signal shown in Figure 2a is used here to illustrate the advantage of EEMD over EMD. The simulated signal is a sine wave of 36 Hz attached by small impulses. Thus, it involves two signal components (b) and (c) shown in Figure 2b and c, respectively. 

**Figure 3:**
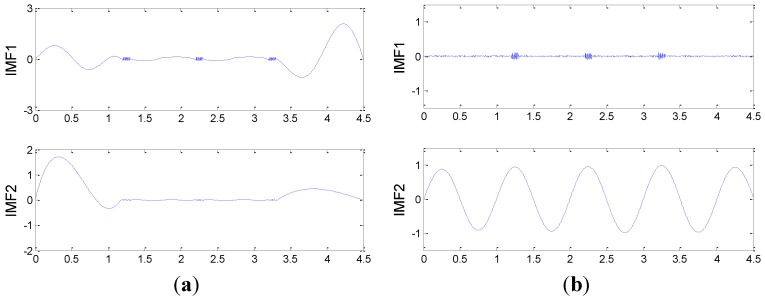
The decomposition result of the simulation signal: (**a**) with EMD; (**b**) with EEMD.

Figure 3a and b show the decomposition results obtained by the EMD and the EEMD methods, respectively. In Figure 3a, the first and the second intrinsic mode functions (IMF1 and IMF2) correspond to the components (b) and (c) in the simulated signal. It is evident that these two completely different signal components are not separated and are still distributed in one IMF. In Figure 3b, IMF1 corresponds to the high-frequency impulsive signal components and IMF2 corresponds to the low-frequency sine wave. Thus, the EEMD method has better decomposition performance and would be used to decompose the raw vibration signals collected from the gearboxes, so that the signals of interest can be well extracted from the raw signals for the following feature extraction. After applying the EEMD method to raw signals and analyzing its spectral features, the first three IMFs obtained in the decomposition of each raw vibration signal are considered in the process of the following feature extraction.

### 3.2. Statistical Feature Extraction Based on DET

For the raw vibration signal collected from a healthy or a faulty gearbox, the 14 time-domain statistical characteristics and 13 frequency-domain statistical characteristics, for a total of 27 feature parameters are selected from each decomposed IMF. More details on the expressions of these features can be referred to in [29]. For each of three IMFs, 81 features (3 × 27) form a feature matrix for each raw vibration signal. 

However, the constructed feature matrix is redundant. Not all the extracted features have the same contributions to faults/damage level classification, and the irrelevant or redundant features may decrease the classification efficiency and increase the computation burden. To further improve the classification accuracy and reduce the dimension of features, the distance evaluation technique (DET) is used to select the dominant features that can well represent the fault characteristics hidden in the raw vibration signal. The main idea for this is to select the most effective features that can well represent the fault features from the entire feature set, *i*81 features. For more details of the DET, please refer to [30].

## 4. Strategy

**Figure 4:**
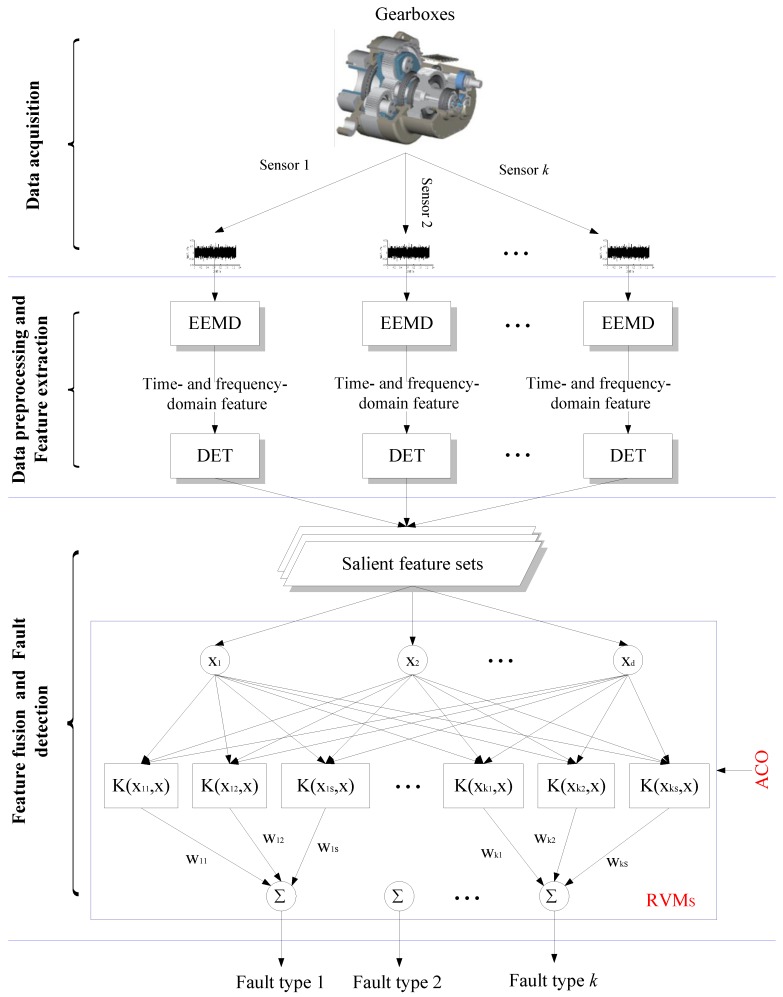
Multi-sensor data fusion strategy of the gearbox fault detection.

In essence, gearbox fault detection based on multi-sensor data fusion is approximately divided into several steps: signal preprocessing to extract the feature vector, feature vector evaluation, and classification according to the evaluated fault feature vector. Therefore, a novel intelligent hybrid strategy, based on EEMD, DET and ACO-RVM, for gearboxes fault detection is presented in this study. The overall process of the proposed data fusion method is illustrated in Figure 4. The main steps are described as follows:

***Step* 1**: Collect vibration signals from different sensors mounted on healthy and faulty gearboxes.
***Step* 2**: Determine the appropriate interval time, partition the collected data of various states into segments (for instance the collected data are partitioned into $T_{S}/t_{I}$ parts as their sampling time is $T_{S}$ and interval time is $t_{I}$).
***Step* 3**: Preprocess each segment by EEMD and obtain their IMFs. Calculate 27 statistic feature including time- and frequency-domain features of the first three IMFs, and the DET is used to select dominant features for each data segment. All segments are fused as samples that are further divided into two subsets, the training samples and testing samples.
***Step* 4**: Using the training samples, the width factor of RVM is optimized by the ACO algorithm according to the procedure described in Section 2.2.
***Step* 5**: Test the well trained RVM to make decision. As a result, the operating conditions of the tested gearbox can be determined.

## 5. Case Studies

To verify the effectiveness of the proposed RVM-based multi-sensor data fusion method, two kinds of gearboxes, the bevel gearbox and the planetary gearbox, are used in the experiments and the corresponding vibration data were collected. In the RBF kernel-based RVM, the width coefficient γ is adjustable, which influences the classification performance of the RVM. The best way is to optimize the parameter γ and retrain the RVM for every data subset. The parameter γ selection using ACO is shown in Figure 1. Furthermore, a comparison between the parameter estimation of the RVM classifier based on the CV [2^−10^, 2^10^] and the ACO are made in this section.

The datasets of two case studies are detailed in Table 1, in which the dataset A comes from *Case* 1: *Bevel gearbox fault detection*, and the dataset B comes from *Case* 2: *Planetary gearbox fault detection*, the proposed RVM-based classifier is performed on a datasets containing different fault severities. The data subset has 50 samples: 30 samples form the training set and the remaining 20 samples are the testing set. 

**Table 1:** The data sets for defect and severity classification.

Dataset	Number of Training Samples	Number of Testing Samples	Length of Each Sample	Condition
A	30	20	2048	normal
	30	20	2048	worn tooth
	30	20	2048	broken tooth
	30	20	2048	missing teeth
B	30	20	4096	cracked tooth
	30	20	4096	pitted tooth
	30	20	4096	chipped tooth
	30	20	4096	missing tooth

### 5.1. Case 1: Bevel Gearbox Fault Detection

#### 5.1.1. Experimental Systems and Data Acquisition

**Figure 5:**
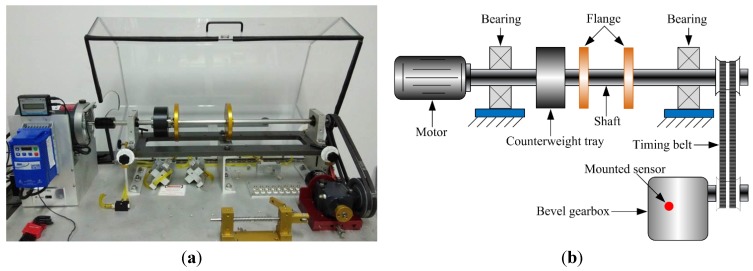
(**a**)The experiment rig of gearbox; (**b**) Sketch map of the gearbox.

**Figure 6:**
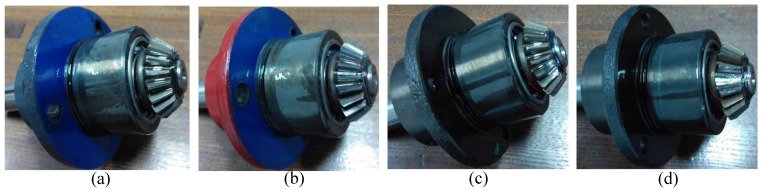
The three bevel gears with different damages: (**a**) normal state; (**b**) worn tooth; (**c**) broken tooth; (**d**) missing teeth.

**Figure 7:**
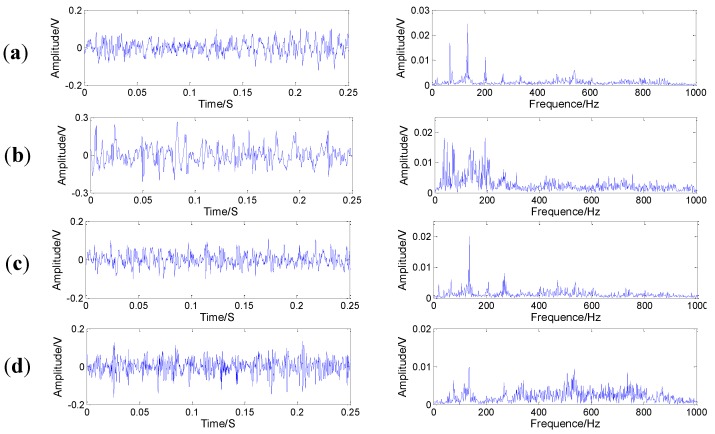
Original time waveforms and their corresponding spectrums of bevel gears in four conditions: (**a**) normal state; (**b**) worn tooth; (**c**) broken tooth; (**d**) missing teeth.

Figure 5 shows the structure diagram of the test rig, which consists of a AC motor, a speed load system, two bearings, bevel gearbox, *etc*. The bevel gearbox is driven by the AC motor and coupled with rub belts. Sensor was mounted on the top of the gearbox. The vibration signals are obtained every 3 min by an eight-channel DAQ, and the speed of the AC motor is 1800 r/min. The sampling frequency is 20 kHz. Specification of the pair of bevel gear is listed in Table 2. In this experiment, four gears with different states are shown in Figure 6, including, a healthy gear, a gear with worn tooth, a gear with broken tooth, and a gear with missing teeth, in which the defects are circled in red. Figure 7 shows the collected temporal waveforms and their corresponding frequency spectra for these four gearboxes.

**Table 2:** Specifications of the bevel gearbox.

Material	Steel/Steel
Number of teeth (*z*/*z*_1_)	27/18
Pressure angle (°)	20
Big gear pitch diameter (inch)	1.6875
Small gear pitch diameter (inch)	1.125
Big gear contact Angle (°)	56°19’
Small gear contact Angle (°)	33°41’

#### 5.1.2. Experimental Results and Analysis

**Figure 8:**
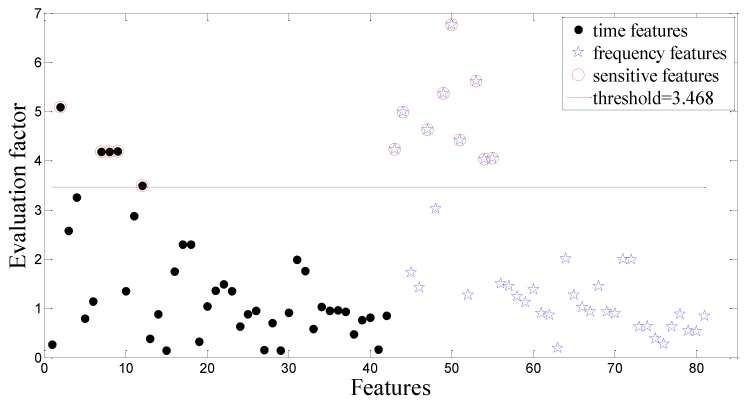
The evaluate factor of all the extracted features in the bevel gearbox test.

Following the flow chart shown in Figure 4, the collected vibration signals from the dataset A are decomposed by the EEMD, and 27 time- and frequency-domain features are extracted from the first three IMFs (the most important information of the vibration signal is included in high-frequency bands). After applying the DET to 81 features obtained from one raw signal, 14 sensitive features are selected and highlighted in red circles shown in Figure 8. Finally, these 14 dominant features as the newly generating feature vector are input of two classifiers based on the CV-RVM and the ACO-RVM, respectively. These two classifiers are individually trained and tested to determine the states of the tested gearbox. 

**Figure 9:**
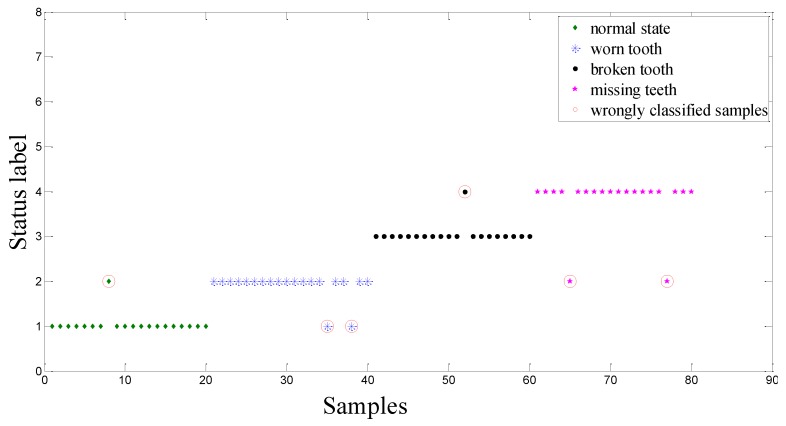
Classification result based on CV-RVM classifier in the bevel gearbox test.

**Figure 10:**
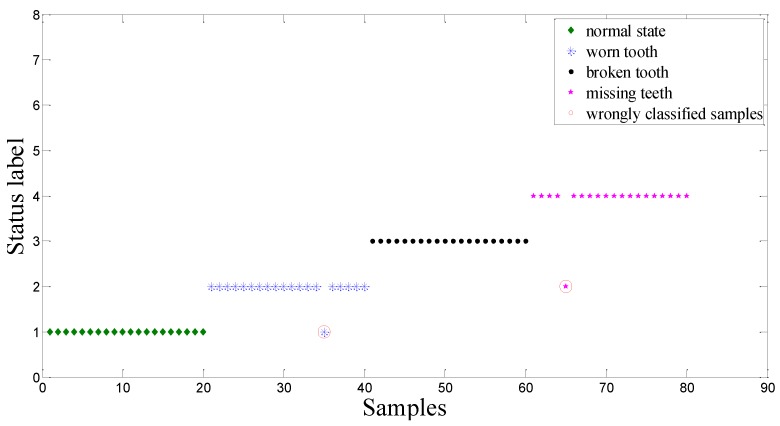
Classification result based on ACO-RVM classifier in the bevel gearbox test.

The detection results using the CV-RVM are shown in Figure 9. 7 misclassified testing samples are circled in red. As for the proposed ACO-RVM classifier, the testing results are shown in Figure 10. As shown in this figure, only 2 testing samples are misclassified and circled in red. 

**Figure 11:**
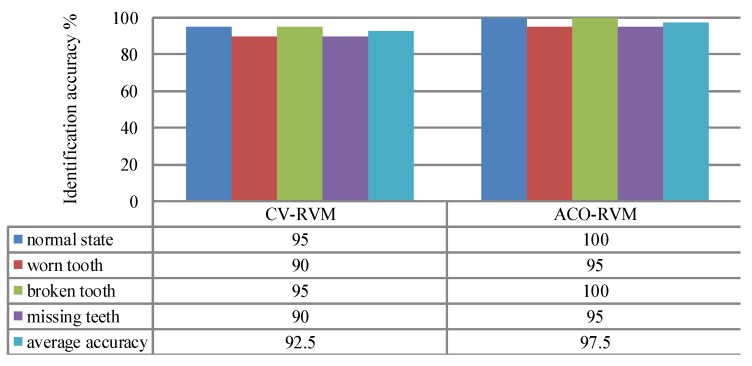
Comparisons of identification accuracy between the CV-RVM classifier and the ACO-RVM classifier in the bevel gearbox test. * The parameter *γ* of the RVM based on the CV and the ACO are denoted by *γ* = 150.11 and *γ* = 2.74, respectively.

Comparisons of identification accuracy (represented by the percentage between samples of correctly identifying the fault modes or defect levels and all tested samples, and a larger value is preferable) between the CV-RVM classifier and the ACO-RVM classifier is shown in Figure 11. When using the CV-RVM classifier, the classification accuracies for gearboxes with the normal state, worn tooth, broken tooth and missing teeth are 95%, 90%, 95% and 90%, respectively. Overall average classification accuracy is 92.5%. When using the ACO-RVM classifier, for the gearboxes with normal state and broken tooth, the classification accuracies are high, 100%. It means that all tested samples are correctly classified. The classification accuracies for the gearboxes with worn tooth and missing teeth are also the same, 95%. The overall average classification accuracy is 97.5%. Compared with CV-RVM classifier, the proposed ACO-RVM classifier improves the recognition accuracy by 5.0%.

### 5.2. Case 2: Planetary Gearbox Fault Detection

#### 5.2.1. Experimental Systems and Data Acquisition

**Figure 12:**
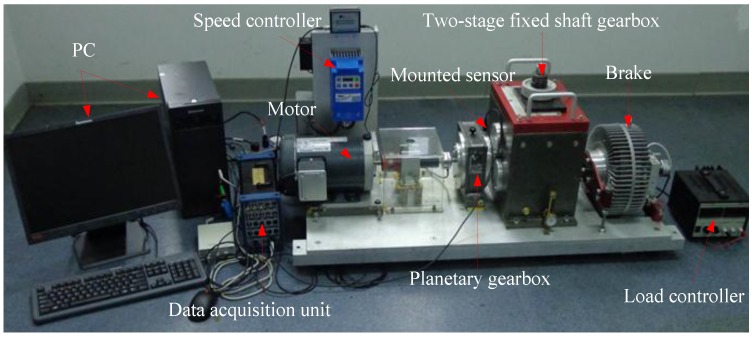
The experiment rig of planetary gearbox.

**Figure 13:**
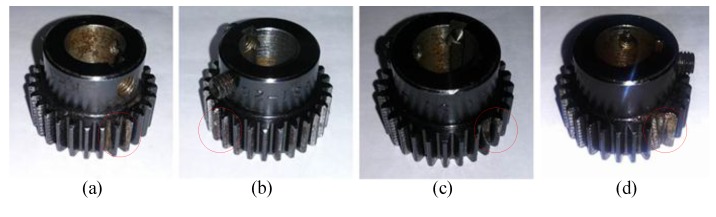
The four sun gears with different damages: (**a**) cracked tooth; (**b**) pitted tooth; (**b**) chipped tooth; (**c**) missing tooth.

**Figure 14:**
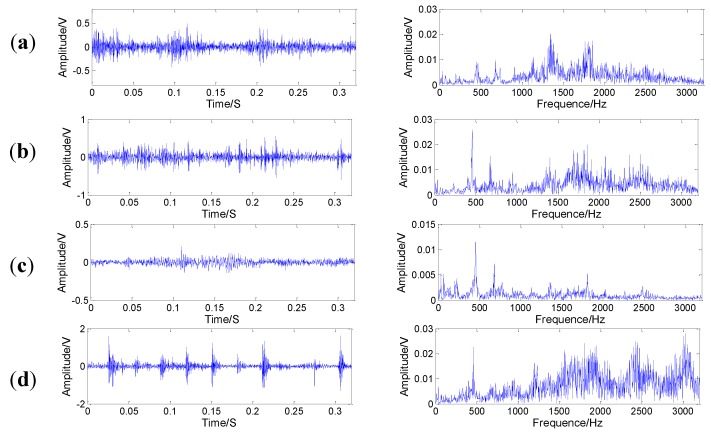
Original time waveforms and their corresponding spectrums of sun gears in four conditions: (**a**) cracked tooth; (**b**) pitted tooth; (**b**) chipped tooth; (**c**) missing tooth.

Figure 12 presents an experimental system of a planetary gearbox test rig, which consists of a AC motor, a magnetic brake, two gearboxes contain a one-stage planetary one and a two-stage fixed-axis one, an NI data acquisition unit, and a PC with the data acquisition software. The one-stage planetary gearbox is the concern in this experiment. In a planetary gearbox, sun gear teeth are subject to faults because their multiple meshes with the planet gears increase the potential for damage [31]. Thus, the four kinds of faults *i.e*., cracked tooth, pitted tooth, chipped tooth and missing tooth on the sun gear are simulated. The pictures of the damaged sun gears are given in Figure 13. Table 3 lists the gear parameters of the planetary gearbox. The sensor is used to capture the vibration signals. Figure 14 shows temporal waveforms and the corresponding frequency spectra of raw vibration signals collected from the tested planetary gearboxes. All vibration signals are measured with a sampling frequency of 20 kHz.

**Table 3:** Specifications of the planetary gearbox.

Material	Steel/Steel
Number of teeth on the sun gear (*z*)	28
Number of teeth on the planet gear (*z*)	36
Number of teeth on the ring gear (*z*)	100
Pressure angle (°)	20
Number of planet gear	4

#### 5.2.2. Experimental Results and Analysis

**Figure 15:**
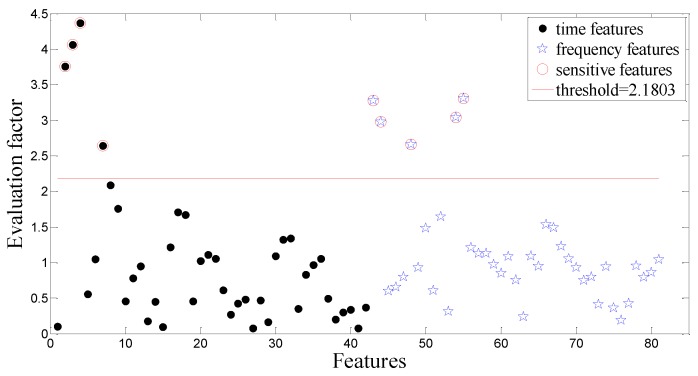
The evaluate factor of all the extracted features in the planetary gearbox test.

**Figure 16:**
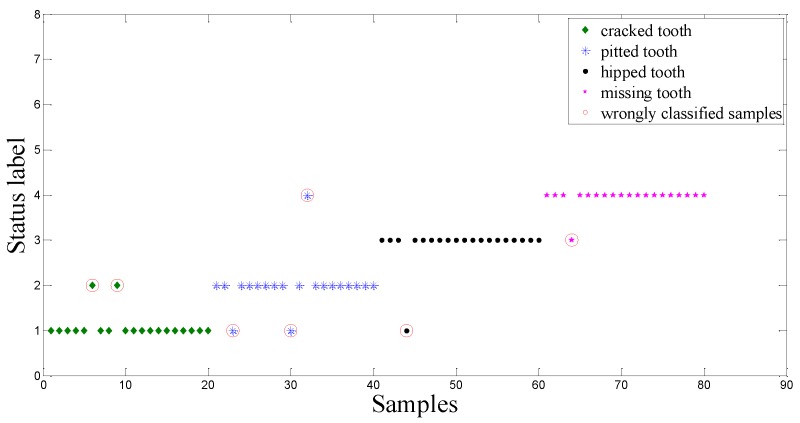
Classification result based on CV-RVM classifier in the planetary gearbox test.

**Figure 17:**
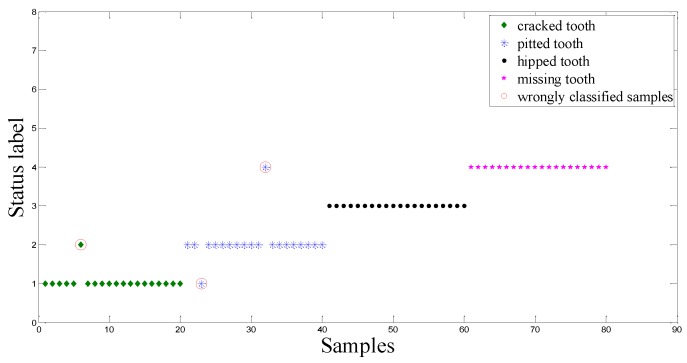
Classification result based on ACO-RVM classifier in the planetary gearbox test.

The above-mentioned intelligent method is employed again to analyze the dataset B. Figure 15, shows the extracted nine sensitive features highlighted by red circles. Using these nine sensitive features as input of the ACO-SVM and the ACO-RVM classifiers, two classifiers are trained and tested. Figure 16 and Figure 17 show the testing results using the CV-RVM classifier and the ACO-RVM classifier, respectively. In Figure 16, 9 testing samples are misclassified by red circles. In Figure 17, only three testing samples are misclassified by red circles.

**Figure 18:**
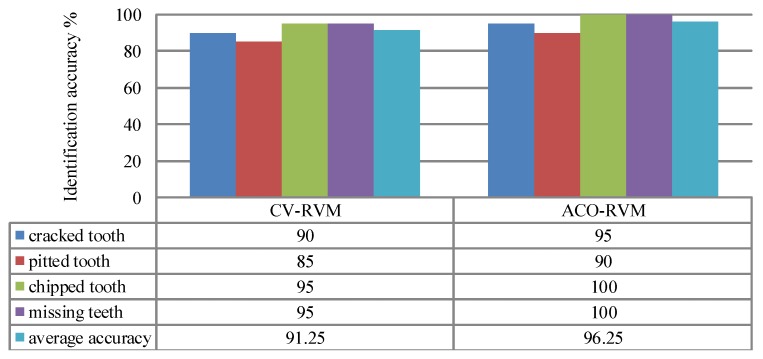
Comparisons of identification accuracy between the CV-RVM classifier and the ACO-RVM classifier in the planetary gearbox test. * The parameter *γ* of the RVM based on the CV and the ACO are denoted by *γ* = 140.74 and *γ* = 2.15 respectively.

The Comparisons of identification accuracy between the CV-RVM classifier and the ACO-RVM classifier for planetary gearbox fault detection is given in Figure 18. When using the CV-RVM method, the classification accuracy for the cracked tooth, pitted tooth, chipped tooth and missing tooth are 90%, 85%, 95% and 95%, respectively. The overall average classification accuracy is 91.25%. 

In Figure 18, the ACO-RVM method proposed in this paper gives the highest classification accuracies for the gearboxes with the chipped tooth and the missing tooth, reach 100%, respectively. The classification accuracies for the gearboxes with the cracked tooth and the pitted tooth are 95% and 90%, respectively. In this experiment, the ACO-RVM classifier possesses a high testing accuracy (96.25%), whereas the CV-RVM classifier shows a relatively poor accuracy (91.25%). Compared with the CV-RVM method, the ACO-RVM method yields fewer misclassified testing samples and greatly improve the classification accuracy.

## 6. Conclusions

This paper presents an intelligent multi-sensor data fusion method using a relevance vector machine based on ant colony optimization (ACO) for gearbox fault detection. In this method, an improved RVM classifier is used as a tool for feature-level data fusion to identify the healthy condition of the tested gearboxes. The ACO is employed to select appropriate parameters for RVM. The proposed method was applied to accomplish preprocessing, feature extraction and fault recognition tasks. The experimental results demonstrate that the proposed ACO-RVM method has higher classification accuracy and better generalization ability than the RVM based on normal the cross-validation (CV) method. It is an effective method for fault detection of rotating machinery working in noisy and complicated environment. 

In addition, in order to obtain more reliable and effective detection result, the optimized RVM will be made toward a higher efficient model beyond current levels, and deeper study on more datasets is needed to perfect the proposed method in future research, and also efforts will be made toward multi-sensors data fusion fault detection in more fields.
